# Kinetics of the chromosome 14 microRNA cluster ortholog and its potential role during placental development in the pregnant mare

**DOI:** 10.1186/s12864-018-5341-2

**Published:** 2018-12-20

**Authors:** Pouya Dini, Peter Daels, Shavahn C. Loux, Alejandro Esteller-Vico, Mariano Carossino, Kirsten E. Scoggin, Barry A. Ball

**Affiliations:** 1 Faculty of Veterinary Medicine, Ghent University, Merelbeke, Belgium; 2 Gluck Equine Research Center, Department of Veterinary Science, University of Kentucky, Lexington, KY USA

**Keywords:** C14MC, microRNA, mRNA, Transcriptome, microRNA-cluster, Human chromosome 14, Pregnancy, Angiogenesis, Equine chromosome 24, Mare

## Abstract

**Background:**

The human chromosome 14 microRNA cluster (C14MC) is a conserved microRNA (miRNA) cluster across eutherian mammals, reported to play an important role in placental development. However, the expression kinetics and function of this cluster in the mammalian placenta are poorly understood. Here, we evaluated the expression kinetics of the equine C24MC, ortholog to the human C14MC, in the chorioallantoic membrane during the course of gestation.

**Results:**

We demonstrated that C24MC-associated miRNAs presented a higher expression level during early stages of pregnancy, followed by a decline later in gestation. Evaluation of one member of C24MC (miR-409-3p) by in situ hybridization demonstrated that its cellular localization predominantly involved the chorion and allantoic epithelium and vascular endothelium. Additionally, expression of predicted target transcripts for C24MC-associated miRNAs was evaluated by RNA sequencing. Expression analysis of a subset of predicted mRNA targets showed a negative correlation with C24MC-associated miRNAs expression levels during gestation, suggesting the reciprocal control of these target transcripts by this miRNA cluster. Predicted functional analysis of these target mRNAs indicated enrichment of biological pathways related to embryonic development, endothelial cell migration and angiogenesis. Expression patterns of selected target mRNAs involved in angiogenesis were confirmed by RT-qPCR.

**Conclusion:**

This is the first report evaluating C24MC kinetics during pregnancy. The findings presented herein suggest that the C24MC may modulate angiogenic transcriptional profiles during placental development in the horse.

**Electronic supplementary material:**

The online version of this article (10.1186/s12864-018-5341-2) contains supplementary material, which is available to authorized users.

## Background

Small non-coding RNAs (ncRNAs) constitute a group of RNAs which do not encode for proteins, but instead modify the expression of protein-coding mRNAs [1]. In plants and animals, microRNAs (miRNA) were one of the first identified families of regulatory ncRNAs [2, 3]. MiRNAs are single-stranded small RNAs, approximately 22 nucleotides in length, that post-transcriptionally modulate protein-coding genes [4]. These ncRNAs regulate numerous cellular processes such as metabolism, cell proliferation, apoptosis, and cell differentiation in almost all cell types. The full or partial complementarity of base pairs between miRNA and their respective target mRNA lead to degradation or blocking the translation of the target mRNAs [4–6].

One of the unique features of miRNAs is that they can be transcribed as clusters. A miRNA cluster is a group of two or more miRNAs which are transcribed from physically adjacent miRNA genes in the same orientation [7, 8]. These miRNA clusters are exclusively or preferentially expressed in a tissue-specific manner [9]. Recent reports have described several miRNA clusters expressed in human placenta, including the chromosome 14 miRNA cluster (C14MC), the chromosome 19 miRNA cluster (C19MC) and the miR-371-3 cluster also located in chromosome 19 [9–12]. The C19MC is exclusively found in primates while C14MC appears to be conserved among all eutherian species studied so far [12]. To date, the expression of miRNA clusters in equine placenta has not been reported.

The chromosome 14 miRNA cluster is one of the largest miRNA clusters transcribed in human and mouse (C12MC) placenta and consists of over 50 miRNAs. This cluster was originally described as two families located within two closely neighboring segments spanning about 40 kb [12–14]. The expression of C14MC has a tissue-specific pattern in mice and in humans [9, 14]. In mice, C12MC is mostly expressed in the head and trunk of the developing embryo and it is restricted to the brain and placental tissues in the adult mice [14]. In healthy humans, expression of the C14MC is predominantly limited to the placenta and other tissues of epithelial origin [9]. The expression of C14MC was also observed in abnormal tissues in humans and mice, as potential tumor suppressor factors in gastric and liver cancers, mainly by inhibiting cell migration and invasion [15–17]. Currently, the tissue-specific expression of this cluster has not been characterized in species other than humans and mice.

While eutherian mammals have a wide diversity in the morphology of their placenta, the orthologous miRNA clusters are conserved without significant structural changes in all sequenced genomes and appear to be maintained in evolution for approximately 100 million years [13]. It has been suggested that the emergence of the C14MC was one of the factors that facilitated evolution of the mammalian placenta and may play important biological roles in placental and embryonic development [13, 18]. For instance, the importance of the murine C12MC, orthologous to C14MC, has been experimentally verified in a mouse model by deletion of this cluster, which led to neonatal lethality [19]. The C12MC-associated miRNA knockout mice showed placental over growth and a defective feto-maternal interface [20, 21]. In contrast, overexpression of C12MC-associated miRNAs was associated with growth retardation and postnatal lethality [22]. Although the C14MC has been reported to play an important role in placental development, its expression kinetics and its functions are poorly understood. Morales-Prieto et al. described the expression of miRNAs within C14MC in human trophoblast cells and showed a significant decrease in the expression levels of this cluster between first to third trimester of pregnancy, suggesting a strong decline in the expression of C14MC with advancing gestational age in humans [11]. Gu et al. also demonstrated that expression of miRNAs within C14MC was significantly upregulated in the first trimester human placenta in comparison to postpartum placental samples [23]. These studies investigated the expression of C14MC at only two time-points due to limited sample availability, so there is minimal information on the kinetics of this cluster throughout pregnancy. Characterizing the expression pattern of miRNAs during physiological and pathological conditions is an important first step to investigate the function of this critical miRNA cluster.

We hypothesized that equine C24MC-associated miRNAs, the ortholog of human C14MC, have a differential expression pattern in chorioallantoic membrane (CAM) during different stages of gestation. In the present study, we determined the expression pattern of equine C24MC-associated miRNAs in CAM during pregnancy. We further analyzed the predicted functions of this cluster by generating a corresponding RNA-sequencing dataset from corresponding CAM. Additionally, we analyzed the cellular localization of one member of this cluster (eca-miR-409-3p) throughout gestation in equine CAM by in situ hybridization (ISH). This study identified that the expression of C24MC-associated miRNAs is downregulated during the course of gestation and determined the cellular localization of one of its members. Furthermore, we showed the presence of a negative correlation between the expression profile of these miRNAs and that of their putative mRNA targets, predicted to be functionally involved in embryonic development, endothelial cell migration and angiogenesis. Further studies to characterize the functions of each member of this miRNA cluster during placental development are warranted.

## Methods

### Animal use

The horses (*Equus caballus*) used in this study were mixed-breed ranging between 250 and 550 kg and four to sixteen years of age. Mares were born and raised at University of Kentucky research farm and housed on pasture with ad libitum grass hay. Gestational age was determined based on the day of ovulation (day 0).

### CAM collection and preparation

Samples of CAM (*n* = 29) were collected from pregnant mares at 45 days (45d, *n* = 9), four months (4mo, *n* = 7), six months (6mo, *n* = 4) and ten months of gestation (10mo, *n* = 6), as well as from the CAM after normal parturition (*n* = 3, postpartum). For collection of CAM samples (except for CAM at 45d and postpartum), the uterus of pregnant mares was recovered immediately after euthanasia (using a barbiturate overdose following the American Veterinary Medical Association [AVMA] guidelines for the euthanasia of animals), the CAM was carefully separated from the endometrium, and full thickness CAM were collected from the body of the placenta. The CAM samples at 45d were retrieved from conceptuses collected by uterine lavage. The postpartum samples were collected immediately after normal parturition.

**Fig. 1:**
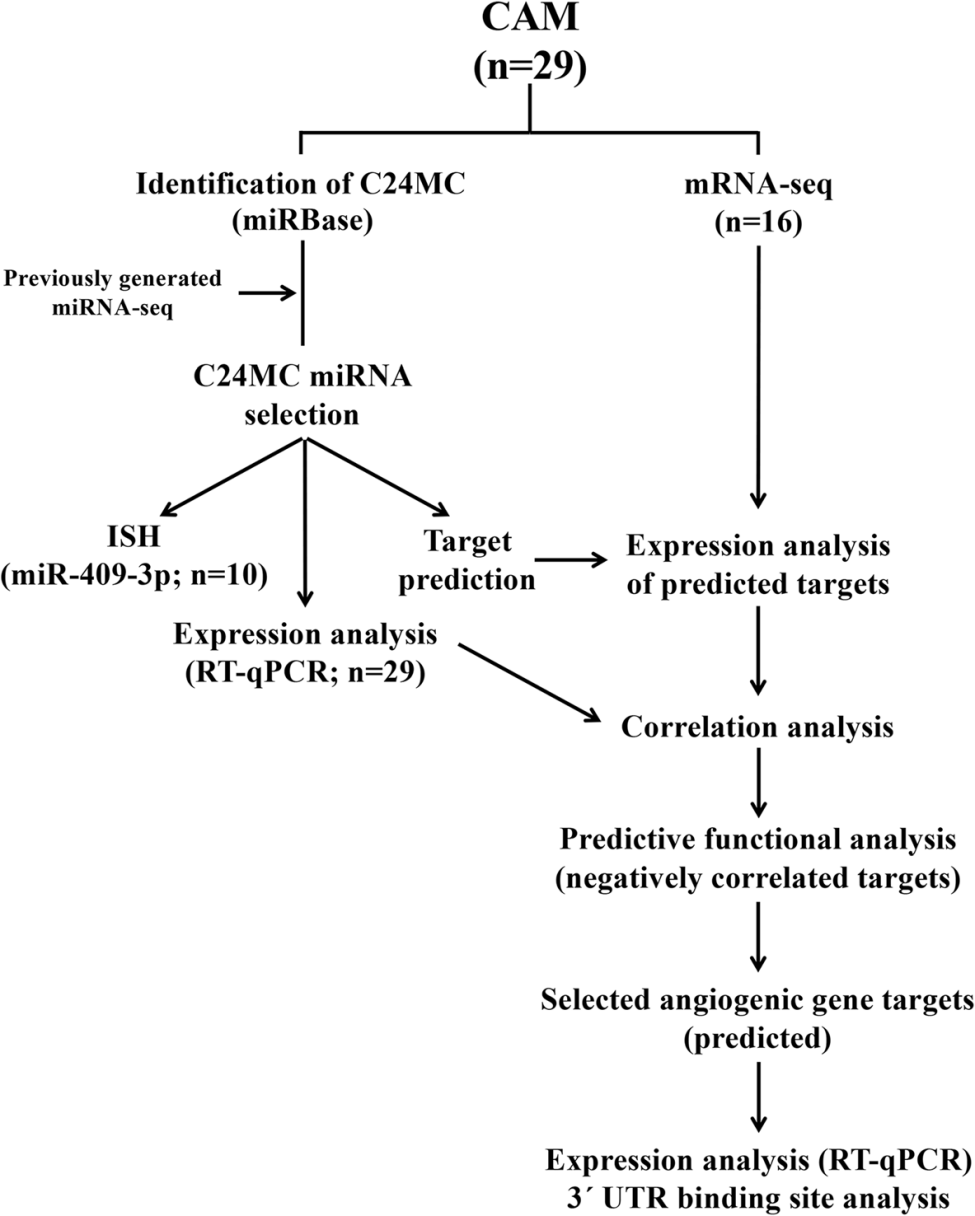
Analysis workflow carried out in this study. CAM, chorioallantoic membrane; C24MC, chromosome 24 microRNA cluster; mRNA-seq, mRNA sequencing; miRNA-seq, microRNA sequencing; ISH, in situ hybridization; RT-qPCR, UTR, untranslated region

Collected CAM samples were stored overnight at 4 °C in RNA*later*™ (Life Technologies, Carlsbad, CA) and then stored at − 80 °C until further processed. The remaining tissue samples were fixed in 10% formaldehyde for 24 h, transferred to 70% methanol and paraffin embedded following standard histological procedures [24]. Histological sections were stained with hematoxylin and eosin following routine procedures and assessed to confirm normal CAM without any contamination from endometrium. The study overview is presented in Fig. 1.

### MiRNA extraction, cDNA synthesis and qPCR

CAM samples were thawed on ice and total RNA was extracted with Trizol™ (Life Technologies, Carlsbad, CA) following the manufacturer’s recommendations. Total RNA concentration was measured using a NanoDrop DP-1000 spectrophotometer (ThermoFisher Scientific, Waltham, MA).

Complementary DNA synthesis was carried out using the miScript II RT Kit (Qiagen, Hilden, Germany). The reaction carried out in 20 μL reverse transcription reaction mixture consisted of 2 μL of extracted RNA (containing 400 ng of RNA), 4 μL of miScript HiSpec buffer, 2 μL of miScript Nucleics mix, 10 μL of RNase-free water and 2 μL of miScript Reverse Transcriptase mix. Three μL of cDNA product from each sample were combined to make the pooled CAM sample. Pooled samples were only used as RT-qPCR inter-plate controls as previously described [25].

The expression of mature miRNAs was determined by RT-qPCR using the miScript SYBR Green PCR kit (Qiagen, Hilden, Germany) with the miScript Universal Primer along with miRNA-specific primers according to the manufacturer’s guidelines. The reaction consisted of 1 ng (1 μl) of cDNA added to a reaction volume (9 μl) containing 2X QuantiTect SYBR® Green PCR Master Mix (5 μl), 10X miScript Universal Primer (1 μl), assay specific primers (1 μl of a 5 μM stock, final concentration 0.5 μM), and RNase free water (2 μl). The cycling conditions included an initial PCR activation step (95 °C for 15 min), followed by 40 cycles of denaturation at 94 °C for 15 s, annealing at 55 °C for 30 s, and extension at 70 °C for 30 s. A DNA melting curve was generated to discriminate between specific and non-specific amplification products. Primers were designed using miRprimer software (version 2.0) for 30 candidate miRNAs [26]. The candidate miRNAs were selected in three steps:

(i) Based on prediction of C24MC in miRBase.org (miRbase.v.21) (EquCab2.0), 46 distinct miRNAs are included in this cluster with an inter-miRNA distance of less than 10 kb (EquCab2.0). The comparative list of C14MC in domestic animals is presented in the Additional file 1: Table S1.
(ii) Based on our previously generated Illumina miRNA-sequencing dataset of equine CAM, we narrowed the predicted miRNA list (Gene Expression Omnibus [GEO, NCBI, NIH] Accession GSE113142). Presence of all the predicted C24MC-associated miRNAs (based on miRBase.org) were confirmed in the CAM with the miRNA-sequencing dataset from 4mo (*n* = 3), 10mo (*n* = 3) and postpartum (*n* = 3). Information about miRNA-sequencing, mapping, normalization and statistical analysis are provided in the supplementary section (Additional file 2 and Additional file 3: Table S3 and Additional file 4: Table S4). Primers were designed for the 11 miRNAs that showed significant differences between the examined time points (*P* < 0.05) and for the 17 most abundant miRNAs (primers for a total of 28 miRNA targets were designed).
(iii) Based on a literature search, two additional miRNAs (hsa-miR-154 and hsa-miR-1247) were added to the list of candidate miRNAs [11, 12, 23]. Hsa-miR-154 belongs to the human C14MC and the equine orthologous miRNA has not been reported in miRBase database even though it was expressed in our miRNA-sequencing dataset. Similarly, hsa-miR-1247 was added to the candidate miRNAs since it has been jointly studied with the C14MC even though it is expressed from the opposite DNA strand [14]. It has been shown that the expression of these miRNAs change significantly during both normal and abnormal pregnancy conditions in human [27, 28].

The list of miRNA candidates and primer sequences are provided in Table 1. Primer efficiency was verified on the pooled samples. Primers with C_T_ values < 35 that did not produce primer-dimers were used for further experimentation. Otherwise, primers were re-designed and re-tested. Eca-miR-106a, eca-miR-8908a and eca-miR-369-5p were used as reference genes [25]. Real-Time qPCR was performed in triplicate for all samples, and geometric mean for the triplicate was calculated and used for further analysis [25, 29–31]. PCR efficiencies were calculated using LinRegPCR (version 2012.0) (http://www.hartfaalcentrum.nl) to ensure that all primers resulted in PCR efficiencies between 1.8 and 2.1.

**Table 1:** List of selected C24MC-associated miRNAs evaluated by RT-qPCR and their respective primer sequences

MiRNA ID	Accession ID^a^	Mature sequence	Forward Primer
eca-miR-127-5p	MIMAT0004604	cugaagcucagagggcucugau	ctgaagctcagagggct
eca-miR-134-5p	MIMAT0013127	ugugacugguugaccagagggg	gcagtgtgactggttgac
eca-miR-323-3p	MIMAT0013132	cacauuacacggucgaccucu	gcagcacattacacggt
eca-miR-323-5p	MIMAT0013131	aggugguccguggcgcguucgc	ccgtggcgcgtt
eca-miR-369-3p	MIMAT0013141	aauaauacaugguugaucuuu	cagcgcagaataatacatggt
eca-miR-370-3p	MIMAT0013142	gccugcugggguggaaccuggu	cctgctggggtgga
eca-miR-370-5p	MIMAT0026483	caggucacgucucugcaguuac	cagcaggtcacgtctct
eca-miR-379-5p	MIMAT0013147	ugguagacuauggaacguagg	cagtggtagactatggaacg
eca-miR-382-5p	MIMAT0013150	gaaguuguucgugguggauucg	aggaagttgttcgtggtg
eca-miR-3958-3p	MIMAT0034486	cagauauugcacgguugaucucuu	gcagatattgcacggttga
eca-miR-3958-5p	MIMAT0019275	agguuguccgugauguauuugc	agaggttgtccgtgatgt
eca-miR-409-3p	MIMAT0013152	gaauguugcucggugaaccccu	aggaatgttgctcggtga
eca-miR-411-3p	MIMAT0013154	uauguaacacgguccacuaacc	cagtatgtaacacggtccac
eca-miR-411-5p	MIMAT0003329	uaguagaccguauagcguacg	cagtagtagaccgtatagcgt
eca-miR-412-3p	MIMAT0013155	uucaccugguccacuagccg	gcagttcacctggtcca
eca-miR-412-5p	MIMAT0026557	uggucgaccaguuggaaaguaau	cagtggtcgaccagttg
eca-miR-432-5p	MIMAT0013157	ucuuggaguaggucauugggugg	cagtcttggagtaggtcattg
eca-miR-433-3p	MIMAT0013158	aucaugaugggcuccucggugu	catgatgggctcctcg
eca-miR-485-3p	MIMAT0013160	gucauacacggcucuccucucu	gcaggtcatacacggct
eca-miR-485-5p	MIMAT0013159	agaggcuggccgugaugaauuc	ggctggccgtgatga
eca-miR-487a-5p	MIMAT0026559	gugguuaucccugcuguguucg	caggtggttatccctgct
eca-miR-487b-3p	MIMAT0013162	aaucguacagggucauccacuu	cagaatcgtacagggtcatc
eca-miR-493b-5p	MIMAT0002813	uuguacaugguaggcuuucauu	gcgcagttgtacatggtag
eca-miR-543-3p	MIMAT0013169	aaacauucgcggugcacuucuu	gcagaaacattcgcggtg
hsa-miR-1247-3p	MIMAT0022721	ccccgggaacgucgagacuggagc	cgggaacgtcgagac
hsa-miR-154-5p	MIMAT0000452	uagguuauccguguugccuucg	gcagtaggttatccgtgttg

^a^mirbase.org; Release 21

Primers were designed using miRprimer software (version 2.0)

### Total RNA isolation, library preparation and RNA-sequencing (RNA-seq)

A subset of CAM samples from 45d, 4mo, 6mo, and 10mo (*n* = 4, at each time point) were thawed on ice and total RNA was extracted using the RNeasy Mini Kit (Qiagen) per manufacturer’s instructions. After extraction, RNA was analyzed by NanoDrop® (Thermo Fisher Scientific) and Bioanalyzer® (Agilent, Santa Clara, CA, USA) to evaluate concentration, purity and integrity of recovered RNA. All samples had a 230/260 ratio > 1.8, a 260/280 ratio > 2.0 and an RNA integrity number > 8.0 (mean ± STD = 9.2 ± 0.4).

Library preparation was performed using the TruSeq Stranded mRNA Sample Prep Kit (Illumina, San Diego, CA, USA) per manufacturer’s instructions. The adapter for Read 1 was AGATCGGAAGAGCACACGTCTGAACTCCAGTCACNNNNNNATCTCGTATGCCGTCTTCTGCTTG, with NNNNNN being the index sequence. The read 2 adapter was AGATCGGAAGAGCGTCGTGTAGGGAAAGAGTGTAGATCTCGGTGGTCGCCGTATCAT. The libraries were quantified by qPCR. Sequencing was performed on a HiSeq 4000 (Illumina) using a HiSeq 4000 sequencing kit version 1, generating 150 bp paired-end reads. FASTQ files were generated and demultiplexed using bcl2fastq v2.17.1.14 Conversion Software (Illumina).

For RNA-seq data analysis, the reads were initially trimmed for adapters and quality using TrimGalore Version 0.4.4 (Babraham Bioinformatics; www.bioinformatics.babraham.ac.uk), then trimmed reads were aligned to the horse reference genome (EquCab2.1) using STAR (Release 2.5.2b) [32], and annotated to the equine reference transcriptome available in Ensembl database (EquCab2.88; www.ensembl.org) using Cufflinks (Release 2.2.1; http://cole-trapnell-lab.github.io/cufflinks/) [33]. Differential expression analysis was performed on the normalized read count (FPKM; fragments per kilobase of exon per million mapped reads) [34].

### MiRNA target prediction

To assess pathways that might be affected by the C24MC-associated miRNAs, predicted targets of differentially expressed miRNAs were assessed using Ingenuity Pathway Analysis (IPA; Qiagen, Redwood City, CA, USA; Release December 2017). The miRNA and mRNA pairing was performed using the miRNA expression values (evaluated by RT-qPCR) and normalized read counts (RNA-seq dataset) using IPA’s microRNA Target Filter, respectively. This allows predicting the function for the miRNAs according to the provided datasets (RNA-seq) (www.ingenuity.com/products/IPA/microRNA.html). Only target mRNAs that were previously (experimentally) confirmed or predicted with high confidence (IPA scoring system) were selected as putative targets. In addition to IPA predicted targets, putative targets were also identified using miRDB (http://mirdb.org), and mRNAs with a target prediction score (TPS) of ≥80 were added to the putative target list [35].

Since miRNAs mainly down regulate their target mRNA expression [36, 37], those mRNAs in the RNA-seq data set which exhibited the reverse pattern of expression when compared to the miRNA expression pattern were selected for further analysis. The negative correlation (*r* ≤ − 0.5, *P* < 0.05) between the expression pattern of putative target mRNAs and that of their corresponding miRNA was analyzed during gestation (45d, 4mo, 6mo, 10mo; significance set at *P*-value < 0.05) and those target mRNAs presenting a significant negative correlation with the expression pattern of their corresponding miRNA were selected for canonical pathway and functional analysis [38]. The canonical pathways and functional analysis for the target mRNAs were performed using IPA and biological functions of the target mRNA were analyzed by Protein ANalysis THrough Evolutionary Relationships Classification System (PANTHER; Release 13.1) ontology classification system [39].

### Confirmation of angiogenic gene expression by reverse transcription and quantitative real-time PCR

RNA was isolated from CAM (*n* = 29) at 45d (*n* = 9), 4mo (*n* = 7), 6mo (*n* = 4) and 10mo (*n* = 6) of gestation, as well as from the CAM after normal parturition (*n* = 3, postpartum) using the RNeasy Mini kit per manufacturer’s instructions, including an on-column DNase treatment. RNA concentration was analyzed by NanoDrop®. All samples had a 230/260 ratio > 1.8 and a 260/280 ratio > 2.0 and RIN values > 8 (mean ± STD = 9.2 ± 0.4).

RNA was reverse-transcribed using TaqMan™ Reverse Transcription Reagents (#4368814; Invitrogen™). The reaction (20 μl) included 10 μl of total RNA (2 μg), 2 μl of 10X RT Buffer, 0.8 μl 25X dNTP Mix (100 mM), 2 μl of 10X RT Random Primers, 1 μl of Reverse Transcriptase, 1 μl of RNase inhibitor and 3.2 μl of nuclease-free water. The reverse transcription reaction was incubated for 10 min at 25 °C, followed by 120 min incubation at 37 °C and a final step at 85 °C for 5 min. cDNA was diluted 1:5 in nuclease-free water and stored at − 20 °C until used.

RT-qPCR was performed using PowerUp™ SYBR™ Green Master Mix (#A25741; Applied Biosystems™) for activin A receptor like type 1 (*ACVLR1)*, AXL receptor tyrosine kinase (*AXL)*, C-X-C motif chemokine ligand 10 (*CXCL10)*, dishevelled segment polarity protein 1 (*DVL1)*, endothelin receptor type A (*ENDRA)*, epsin 2 (*EPN2)*, glucose-6-phosphate dehydrogenase (*G6PD)*, junction adhesion molecule 2 (*JAM2)*, neuropilin 1 (*NRP1)* and TYRO3 protein tyrosine kinase 3 (*TYRO3),* which were predicted in our dataset to be involved in angiogenesis (Primer sequences can be found in Additional file 5: Table S5). Briefly, 1 μl (1 ng) of cDNA was added to a reaction volume (9 μl) containing 2X PowerUp™ SYBR™ Green Master Mix (5 μl), assay-specific forward and reverse primers (0.25 μl of a 20 μM stock, final concentration 0.5 μM for each primer) and RNase-free water (3.5 μl). The cycling conditions included an initial UDG (50 °C for 2 min) and PCR activation steps (95 °C for 2 min) followed by 40 cycles of denaturation at 95 °C for 15 s and annealing/extension at 60 °C for 1 min. Melt curve analysis was performed to check for non-specific amplifications along with the inclusion of non-template controls. Reactions were performed in duplicate and *GAPDH, ACTB* and *GUSB* were used as housekeeping genes [40, 41]. PCR efficiencies were calculated using LinRegPCR (version 2012.0) (http://www.hartfaalcentrum.nl) to ensure that all primers resulted in PCR efficiencies between 1.8 and 2.1.

### Analysis of putative miRNA:mRNA binding sites for selected angiogenic genes

For further analysis of potential miRNA:mRNA interactions, identification of the miRNA binding site at the 3’ UTR of the selected equine angiogenic genes was performed. 3’ UTR sequences of the genes which were shown to be involved in angiogenesis in our dataset were retrieved from NCBI and the binding site was identified using IntaRNA 2.0 (Freiburg RNA Tools, Universität Freiburg, Germany) [42, 43]. The free energy (ΔG) of the predicted miRNA:mRNA interaction was estimated using the RNAhybrid tool (Universität Bielefeld, Germany) [44].

### MiRNA localization by in situ hybridization

The expression pattern of eca-miR-409-3p was further investigated by chromogenic ISH using a dual digoxigenin (DIG)-labeled LNA™ probe specific to hsa-miR-409-3p (610701-360; miRCURY LNA™, Exiqon, Vedbaek, Denmark). In addition, a dual DIG-labeled LNA™ probe specific to U6 snRNA (#699002-360; Exiqon) and a scrambled miRNA probe (#699003-360; Exiqon) were used as positive and negative controls, respectively. The sample set evaluated included CAM from 45d (*n* = 2), 4 mo (n = 2), 6 mo (n = 2), 10 mo (n = 2), and postpartum (n = 2). The ISH assay was performed as recommended by the manufacturer. Briefly, five-micrometer sections of formalin-fixed, paraffin-embedded tissues were mounted on positively charged Superfrost® Plus slides (Fisher Scientific, Pittsburgh, PA), dried overnight at 37 °C, and subjected to deparaffinization in xylenes followed by rehydration through an ethanol dilution series and 1X phosphate buffered saline. Automated ISH was performed in a Ventana Discovery Ultra (Ventana Medical Systems, Inc. AZ, USA). The tissue sections were digested with 10 μg/ml of proteinase K for 16 min at room temperature and hybridized with the respective probes (diluted to 40 nM for hsa-miR-409-3p and scrambled miRNA probes, or 0.1 nM for U6 snRNA probe in DISCOVERY ISH diluent (Ventana)) for two hours at 50 °C. Stringency washes were performed with RiboWash (Ventana). The bound dual-labeled probe was detected with an alkaline phosphatase-conjugated polyclonal anti-DIG antibody (DISCOVERY anti-DIG AP Multimer, Ventana) after a 15 min blocking step using the DISCOVERY Antibody Block (Ventana), and the signal was detected using nitro blue tetrazolium chloride/5-bromo-4-chloro-3-indolyl-phosphate (NBT-BCIP; ChromoMap Blue Kit, Ventana) as the substrate. The sections were incubated with the substrate for two hours and counterstained using the Red Counterstain II (Ventana) for 8 min. Sections were finally dehydrated in successive alcohols and mounted using permanent mounting medium (Eukitt®, Sigma-Aldrich, St. Louis, MO). ISH signal was evaluated and quantified by two different investigators blinded to gestational age with a Zeiss operative microscope equipped with MicroPublisher 5.0 RTV camera (Q-Imaging, Burnaby, BC, Canada). For quantification purposes, sections were scored as follows: 0, no staining; 1, weak or minimal staining; 2, mild staining; 3, moderate staining; 4, intense or abundant staining.

### Statistical analysis

Delta C_T_ (ΔC_T_) values for CAM samples were calculated where ΔC_T_ = (C_T_ values of miRNA of interest - Geometric mean of the C_T_ values of all three reference miRNAs) [31, 45]. CAM results are presented as -ΔC_T_ (negative ΔC_T_ is more intuitive than ΔC_T_). Graphs were made in JMP (SAS Institute, version 12.1.0) and “***d3heatmap”*** or ***“plot”*** packages in R (version 1.0.136) [46].

Statistical analyses were performed in SAS version 9.2 (SAS Institute Inc., Cary, NC, USA, 2010). Normality of the data (-ΔC_T_, microRNA-read counts, and FPKM) was verified using “***PROC UNIVARIATE”***. Normal quantile transformation was performed to normalize the data [47]. MiRNA expression levels (-ΔC_T_) were compared between pregnancy stages using the mixed model by “***PROC MIXED”***, where stage of pregnancy was set as the fixed effect and animal was set as the random effect [48]. Post hoc analysis was performed using Tukey’s Test with significance set at *P* < 0.05. The correlation between the RT-qPCR results and miRNA-sequencing results was determined using Pearson correlation coefficient performed in the “***Hmisc*”** package in R with significance set to P < 0.05 [49]. The correlation between the expression patterns of each miRNA and its putative target mRNAs was determined using Pearson correlation coefficient during gestation with significance set to *P* < 0.05 (the false discover rate [FDR] was calculated in the “***p.adjust”*** function in R) [50]. Negative ΔC_T_ was calculated for the predicted genes involved in angiogenesis where ΔC_T_ = (C_T_ values of predicted mRNA - Geometric mean of the C_T_ values of all three housekeeping genes). The correlation between target gene’s FPKM and -ΔC_T_ was evaluated using Pearson correlation coefficient during gestation due to the normality of the data. Also, the correlation between miRNAs` - ΔC_T_ and their target-genes` - ΔC_T_ was measured using Pearson correlation coefficient during gestation.

Mean ISH scores between the two independent observers were determined, and mean scores were compared between the different stages of pregnancy using the logistic regression model for overall significance and Chi square test was used for inter-group (stages of pregnancy) comparison. Significance was set at *P* < 0.05. Due to the non-normal distribution of the ISH scores, the correlation between the expression levels of eca-miR-409-3p determined by RT-qPCR and ISH mean scores was calculated by Spearman correlation (ρ) with significance set at *P* < 0.05.

## Results

### Differential expression of C24MC-associated miRNAs in CAM during pregnancy

**Fig. 2:**
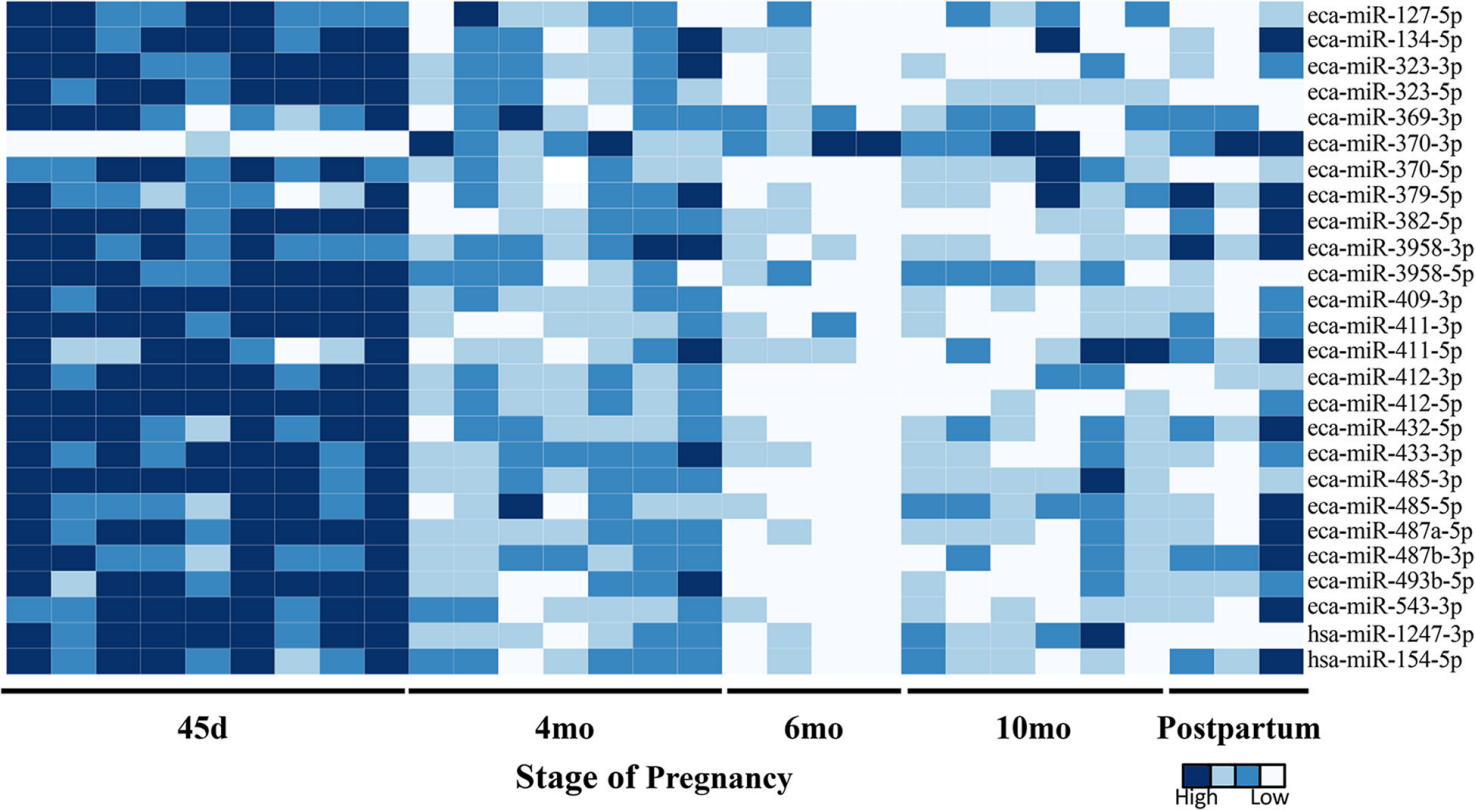
Expression patterns of C24MC-associated miRNAs in CAM during different stages of pregnancy. The heatmap was constructed using -ΔC_T_ values and scaled for each miRNA. The majority of the miRNAs within the cluster (*n* = 21) show a significant higher expression at 45d, with significant downregulation later in gestation. The interactive heatmap can be accessed here. (http://rpubs.com/pouyadini/410338)

The expression of 26 mature miRNAs belonging to the C24MC was quantified in normal equine CAM (*n* = 29) at five different stages of pregnancy (45d, 4mo, 6mo, 10mo, and postpartum) by RT-qPCR (Fig. 2), and the differentially expressed miRNAs (*n* = 23) during the different stages of pregnancy are presented in Table 2 (*P* < 0.05). A total of 21 miRNAs presented a significantly higher expression level at 45d followed by a downregulation later in gestation (Fig. 2 and Table 2). In contrast, eca-miR-370-3p had a lower expression at 45d of gestation in comparison to CAM collected at later stages (Fig. 2 and Table 2). Furthermore, eca-miR-379-5p, eca-miR-432-5p and eca-miR-487b-3p showed a higher expression in postpartum samples in comparison to CAM collected at 6mo. Moreover, eca-miR-3958-3p expression was significantly higher in postpartum compared to CAM from 6mo and 10mo of gestation (Table 2). A significant positive correlation was observed between the normalized read counts derived from miRNA sequencing and -ΔC_T_ values for each of the samples (*r* ranging from 0.71 to 0.9, *P* < 0.002) and the overall correlation coefficient between normalized read counts and -ΔC_T_ was 0.77 (*P* < 0.001). Four miRNAs out of 30 tested miRNAs (eca-miR-136-3p, eca-miR-136-5p, eca-miR-494-3p, and eca-miR-495-3p) were not expressed in > 50% of the samples, with some C_T_ values higher than the accepted range (C_T_ > 35), therefore these miRNAs were excluded from the dataset.

**Table 2:** Differentially expressed C24MC-associated miRNAs during the different stages of pregnancy in equine CAM

miRNA	45 days	4 months	6 months	10 months	PostPartum
	*n* = 9	*n* = 7	*n* = 4	*n* = 6	*n* = 3
eca-miR-127-5p	− 0.18 ± 0.2 ^a^	−1.14 ± 0.22 ^b^	−1.48 ± 0.30 ^b^	−1.21 ± 0.24 ^b^	− 1.65 ± 0.35 ^b^
eca-miR-134-5p	−2.56 ± 0.14 ^a^	−3.24 ± 0.16 ^b^	−3.75 ± 0.21 ^b^	−3.47 ± 0.17 ^b^	− 3.5 ± 0.25 ^b^
eca-miR-323-3p	− 0.25 ± 0.15 ^a^	− 1.27 ± 0.17 ^b^	− 2.18 ± 0.23 ^c^	−1.9 ± 0.19 ^b,c^	−1.55 ± 0.27 ^b,c^
eca-miR-370-3p	2.41 ± 0.16 ^a^	3.38 ± 0.18 ^b^	4.16 ± 0.24 ^b^	3.44 ± 0.2 ^b^	3.97 ± 0.28 ^b^
eca-miR-370-5p	−3.58 ± 0.21 ^a^	−4.6 ± 0.24 ^b^	−5.85 ± 0.32 ^c^	− 4.2 ± 0.26 ^a,b^	−5.18 ± 0.37 ^b,c^
eca-miR-379-5p	3.84 ± 0.3 ^a,b^	3.7 ± 0.34 ^a,b^	2.85 ± 0.45 ^a^	3.66 ± 0.36 ^a,b^	5.42 ± 0.51 ^b^
eca-miR-382-5p	2.13 ± 0.24 ^a^	0.41 ± 0.27 ^b^	− 0.27 ± 0.36 ^b^	−0.09 ± 0.29 ^b^	1.2 ± 0.41 ^a,b^
eca-miR-3958-3p	1 ± 0.21 ^a^	0.78 ± 0.24 ^a,b^	−0.44 ± 0.29 ^c^	−0.2 ± 0.26 ^b,c^	1.22 ± 0.37 ^a^
eca-miR-3958-5p	0.56 ± 0.35 ^a^	− 1.98 ± 0.4 ^b^	−3.56 ± 0.61 ^b^	−1.9 ± 0.43 ^b^	−3.45 ± 0.61 ^b^
eca-miR-409-3p	4.62 ± 0.27 ^a^	2.18 ± 0.31 ^b^	− 0.26 ± 0.41 ^c^	1.48 ± 0.34 ^b^	1.47 ± 0.48 ^b,c^
eca-miR-411-3p	2.89 ± 0.23 ^a^	0.63 ± 0.26 ^b^	−0.04 ± 0.35 ^b^	0.21 ± 0.28 ^b^	0.83 ± 0.4 ^b^
eca-miR-412-3p	−4.57 ± 0.19 ^a^	−6.20 ± 0.22 ^b^	−7.44 ± 0.29 ^c^	−6.57 ± 0.24 ^b,c^	−6.59 ± 0.34 ^b,c^
eca-miR-412-5p	−0.51 ± 0.14 ^a^	−1.98 ± 0.16 ^b^	−2.89 ± 0.21 ^c^	−2.75 ± 0.17 ^c^	−2.30 ± 0.24 ^b,c^
eca-miR-432-5p	2.2 ± 0.29 ^a^	1.09 ± 0.33 ^a,b,c^	−0.34 ± 0.44 ^c^	0.49 ± 0.36 ^b,c^	2.01 ± 0.51 ^a,b^
eca-miR-433-3p	0.84 ± 0.23 ^a^	0.27 ± 0.27 ^a,b^	− 1.27 ± 0.35 ^c^	− 0.67 ± 0.29 ^b,c^	−0.61 ± 0.41 ^b,c^
eca-miR-485-3p	0.62 ± 0.21 ^a^	− 1.18 ± 0.24 ^b^	−2.76 ± 0.32 ^c^	−1.53 ± 0.26 ^b,c^	−2.52 ± 0.37 ^b,c^
eca-miR-485-5p	− 2.42 ± 0.11 ^a^	−2.92 ± 0.13 ^a,b^	−3.43 ± 0.17 ^b^	−2.73 ± 0.14 ^a^	−2.8 ± 0.2 ^a,b^
eca-miR-487a-5p	−1.74 ± 0.14 ^a^	−2.48 ± 0.15 ^b,c^	−3.56 ± 0.21 ^d^	−2.75 ± 0.17 ^d,b^	−2.5 ± 0.24 ^b,c,d^
eca-miR-487b-3p	7.11 ± 0.37 ^a^	5.74 ± 0.42 ^a,b,c^	3.11 ± 0.55 ^d^	4.94 ± 0.45 ^b,c,d^	7.24 ± 0.64 ^a,c^
eca-miR-493b-5p	−3.79 ± 0.18 ^a^	− 4.69 ± 0.21 ^b^	−5.93 ± 0.28 ^c^	−5.26 ± 0.22 ^b,c^	−4.78 ± 0.32 ^a,b,c^
eca-miR-543-3p	1.52 ± 0.2 ^a^	0.07 ± 0.23 ^b,c^	− 1.08 ± 0.3 ^c^	− 0.29 ± 0.24 ^b,c^	−0.17 ± 0.35 ^b,c^
hsa-miR-1247-3p	−4.22 ± 0.16 ^a^	−5.60 ± 0.18 ^b^	−6.83 ± 0.24 ^c^	−5.32 ± 0.19 ^b^	−6.63 ± 0.27 ^c^
hsa-miR-154-5p	1.13 ± 0.2 ^a^	0.31 ± 0.23 ^a,b^	− 0.95 ± 0.3 ^c^	− 0.1 ± 0.24 ^b,c^	0.54 ± 0.35 ^a,b^

Data are presented as Mean ± Standard Error of -ΔC_T_

The expression pattern for each miRNA were compared between timepoints using the mixed model and significant differences between timepoints were determined by subsequent pairwise comparisons. Thus, differing superscripts indicate significant differences between stages of pregnancy with significance set to *P* ≤ 0.05

### C24MC-associated miRNA target prediction

Computational target prediction for miRNAs was performed to identify putative mRNA targets and the expression patterns of predicted target mRNAs present in CAM were evaluated in an Illumina RNA-sequencing dataset generated from a subset of samples (*n* = 16) as indicated under Materials and Methods, subsection 2.4. We generated a total of 723 million reads for 16 samples, and an average of 91.23% (range 89.83 - 92.72%) of reads were uniquely mapped to the reference genome (information about individual sample read count and mapping quality can be found in Additional file 6: Table S6).

**Fig. 3:**
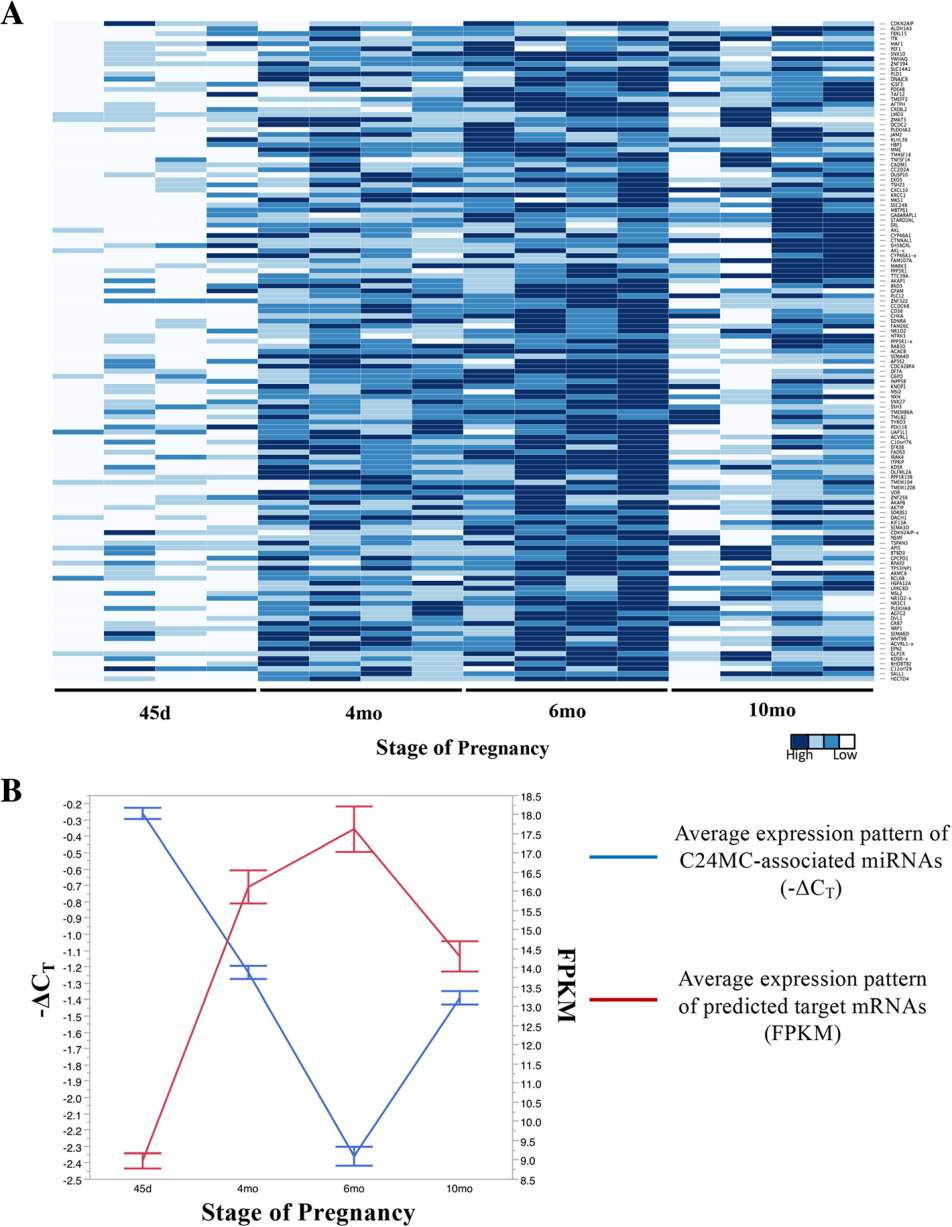
**a**. Expression patterns of mRNAs targets which presented a significant negative correlation to their respective miRNA expression during gestation. The heatmap was constructed using FPKM values and scaled for each mRNA target. The interactive heatmap can be accessed here. (http://rpubs.com/pouyadini/337845​). **b**. Comparative expression analysis between C24MC miRNAs (*n* = 21) and putative mRNA targets (*n* = 130) in equine CAM throughout pregnancy demonstrates a negative correlation in their overall expression pattern (*r* = − 0.78; *P* < 0.05). Correlation was calculated between the average expression pattern of C24MC-associated miRNA (*n* = 26 [no postpartum samples]) and the average expression pattern of predicted target mRNA (*n* = 16). Mean values ± standard error of the means are depicted

A total of 796 target mRNAs were predicted using IPA’s microRNA Target Filter function for the differentially expressed miRNAs that presented a significantly higher expression level at 45d followed by a decline later in the gestation (*n* = 21; Table 3). Additionally, 1179 target genes were predicted for this subset of miRNAs using mirDB. However, 229/1179 predicted genes were not expressed in the associated CAM RNA-seq dataset and were, therefore, excluded from further analysis (Table 3) (the list of putative target mRNAs for each miRNA is available in Additional file 7: Table S7). There was a low agreement between the target lists within the two programs, which is due to the different algorithms used by these programs. Since miRNAs mainly down regulate their target mRNA expression [36, 37], those mRNAs in the RNA-seq data set which exhibited the reverse pattern of expression when compared to the miRNA expression pattern (negative correlation, *r* ≤ − 0.5, *P* < 0.05) were selected for further analysis (Table 4) (*n* = 130 mRNAs, among which seven mRNAs have been predicted for more than one miRNA). High expression levels of C24MC-associated miRNAs in equine CAM collected at 45d are associated with reduced expression levels of the predicted mRNA targets with an inverse relationship as gestational age increases (Fig. 3a and b).

**Table 3:** Number of predicted mRNA targets for a subset of C24MC-associated miRNAs presenting a significantly higher expression level during early stage of pregnancy (45d, *P*-value < 0.05), followed by a decline later in gestation as determined by IPA and mirDB prediction tools

MiRNA ID	IPA	mirDB	Absent in CAM RNA-Seq data^a^	Common between IPA and mirDB
eca-miR-127-5p	49	72	21	5
eca-miR-134-5p	55	43	9	0
eca-miR-154-5p	28	30	6	6
eca-miR-323-3p	15	110	24	4
eca-miR-370-5p	-^#^	62	12	–
eca-miR-382-5p	10	53	10	2
eca-miR-3958-3p	–	–	–	–
eca-miR-3958-5p	–	–	–	–
eca-miR-409-3p	7	61	7	1
eca-miR-411-3p	21	71	12	4
eca-miR-412-3p	65	29	11	1
eca-miR-412-5p	31	2	1	1
eca-miR-432-5p	60	44	10	4
eca-miR-433-3p	78	2	–	–
eca-miR-485-3p	89	160	27	18
eca-miR-485-5p	174	172	23	28
eca-miR-487a-5p	19	53	13	1
eca-miR-487b-3p	19	4	2	1
eca-miR-493b-5p	–	–	–	–
hsa-miR-1247-3p	76	4	3	0
hsa-miR-543-3p	–	207	38	–

^a^ predicted genes with mirDB that were not expressed in our RNA-seq dataset from CAM

# There were no predicted targets for this miRNA

**Table 4:** Correlation analysis between C24MC-associated miRNAs presenting a significantly higher expression level during early stage of pregnancy (45d), followed by a decline later in the gestation, and that of their putative mRNA targets (*n* = 130)

MiRNA ID	Target mRNA	Correlation	*P* value	FDR
eca-miR-127-5p	*MAF1*	−0.5982	0.0144	0.049
	*FBXL15*	− 0.5917	0.0158	0.049
	*ITK*	− 0.5563	0.0252	0.049
	*ZNF394*	− 0.5381	0.0315	0.049
	*PEF1*	−0.5352	0.0326	0.049
	*SNX10*	−0.5212	0.0384	0.049
	*YWHAQ*	−0.5213	0.0384	0.049
	*CDKN2AIP*	−0.5065	0.0453	0.049
	*ALDH1A3*	−0.4986	0.0493	0.049
eca-miR-134-5p	*PLD1*	−0.7269	0.0014	0.003
	*SLC14A1*	−0.5934	0.0154	0.015
eca-miR-323-3p	*CREBL2*	−0.7515	0.0008	0.006
	*AFTPH*	−0.6925	0.0029	0.010
	*DNAJC6*	−0.6175	0.0108	0.017
	*TMEFF2*	−0.6123	0.0117	0.017
	*PDE4B*	−0.6045	0.0131	0.017
	*TAF12*	−0.5981	0.0144	0.017
	*IGSF3*	−0.501	0.048	0.048
eca-miR-370-5p	*ZMAT3*	−0.5327	0.0336	0.049
	*DCDC2*	−0.5266	0.0361	0.049
	*PLEKHA3*	−0.4989	0.0492	0.049
eca-miR-382-5p	*JAM2*	−0.7504	0.0008	0.002
	*KLHL36*	−0.5945	0.0152	0.015
eca-miR-409-3p	*TM4SF18*	−0.7353	0.0012	0.005
	*MME*	−0.6665	0.0048	0.010
	*HBP1*	− 0.6237	0.0098	0.012
	*TNFSF14*	−0.609	0.0123	0.012
eca-miR-411-3p	*SEC24B*	−0.8907	< 0.0001	0.001
	*CXCL10*	−0.7036	0.0024	0.011
	*CC2D2A*	−0.6435	0.0072	0.016
	*MKS1*	−0.6361	0.0081	0.016
	*EXO5*	− 0.6303	0.0089	0.016
	*KRCC1*	−0.6139	0.0114	0.017
	*CADM1*	−0.5907	0.016	0.019
	*TSHZ1*	−0.5881	0.0166	0.019
	*DUSP10*	−0.5092	0.044	0.044
eca-miR-412-3p	*GABARAPL1*	−0.6578	0.0056	0.017
	*STARD3NL*	−0.5598	0.0241	0.036
	*MBTPS1*	−0.5005	0.0483	0.048
eca-miR-412-5p	*SRL*	−0.6407	0.0075	0.008
eca-miR-432-5p	*AXL*	−0.5358	0.0324	0.035
	*CYP46A1*	−0.5283	0.0354	0.035
eca-miR-433-3p	*PPP3R1*	−0.6886	0.0032	0.022
	*CTNNAL1*	−0.6311	0.0088	0.031
	*AXL*	−0.5933	0.0154	0.036
	*FAM107A*	−0.5457	0.0288	0.044
	*SH3BGRL*	−0.5383	0.0315	0.044
	*CYP46A1*	−0.5234	0.0375	0.044
	*MARK3*	−0.5077	0.0447	0.045
eca-miR-485-3p	*CHKA*	−0.797	0.0002	0.003
	*CCDC68*	−0.7795	0.0004	0.003
	*PLCL2*	−0.7534	0.0008	0.004
	*AKAP1*	−0.6916	0.003	0.011
	*CD36*	−0.6645	0.005	0.011
	*NR1D2*	−0.6638	0.005	0.011
	*RAB30*	−0.6624	0.0052	0.011
	*FAM26E*	−0.6531	0.0061	0.011
	*TTC39A*	−0.629	0.009	0.015
	*NTRK3*	−0.6146	0.0113	0.017
	*BRD3*	−0.5751	0.0198	0.027
	*GPAM*	−0.5536	0.0261	0.033
	*ZNF322*	−0.533	0.0335	0.039
	*PPP3R1*	−0.5107	0.0432	0.046
	*EDNRA*	−0.4982	0.0495	0.049
eca-miR-485-5p	*ACACB*	−0.7863	0.0003	0.005
	*SNX27*	−0.7736	0.0004	0.005
	*SSH3*	−0.7668	0.0005	0.005
	*TYRO3*	−0.7498	0.0008	0.005
	*ITPRIP*	−0.752	0.0008	0.005
	*CDC42BPA*	−0.7137	0.0019	0.008
	*FADS3*	−0.706	0.0022	0.008
	*DFFA*	−0.6948	0.0028	0.008
	*KNOP1*	−0.6954	0.0028	0.008
	*MSI2*	−0.6903	0.0031	0.008
	*VDR*	−0.6906	0.0031	0.008
	*OLFML2A*	−0.6713	0.0044	0.011
	*NXN*	−0.6692	0.0046	0.011
	*IRAK4*	−0.6638	0.005	0.011
	*KDSR*	−0.6498	0.0064	0.013
	*INPP5B*	−0.6389	0.0077	0.014
	*C10orf76*	−0.6302	0.0089	0.016
	*PEX11B*	−0.6178	0.0108	0.017
	*TMUB2*	−0.6162	0.011	0.017
	*EFR3B*	−0.5739	0.0201	0.030
	*TMEM86A*	−0.5483	0.0279	0.039
	*G6PD*	−0.5467	0.0284	0.039
	*TMEM104*	−0.5282	0.0355	0.045
	*TMEM120B*	−0.5276	0.0357	0.045
	*ACVRL1*	−0.5224	0.0379	0.045
	*AP3S2*	−0.5101	0.0435	0.048
	*PPP1R13B*	−0.5101	0.0435	0.048
	*ZNF256*	−0.5015	0.0478	0.049
	*SEMA4D*	−0.5011	0.048	0.049
	*UAP1L1*	−0.4978	0.0497	0.049
eca-miR-487a-5p	*TSPAN3*	−0.7604	0.0006	0.005
	*KIF13A*	−0.738	0.0011	0.005
	*SEMA3D*	−0.678	0.0039	0.009
	*NSMF*	−0.6753	0.0041	0.009
	*CDKN2AIP*	−0.5968	0.0147	0.026
	*SORBS1*	−0.5357	0.0325	0.042
	*AKAP6*	−0.5302	0.0346	0.042
	*DACH1*	−0.5236	0.0374	0.042
	*AKTIP*	−0.4985	0.0494	0.049
eca-miR-543-3p	*NR3C1*	−0.6944	0.0028	0.024
	*RPAP2*	−0.6608	0.0053	0.024
	*NR1D2*	−0.6509	0.0063	0.024
	*GPCPD1*	−0.6264	0.0094	0.024
	*BTBD3*	−0.6199	0.0104	0.024
	*LRRC8D*	−0.6084	0.0124	0.024
	*MSL2*	−0.5954	0.015	0.024
	*TP53INP1*	−0.5911	0.0159	0.024
	*HSPA12A*	−0.5872	0.0168	0.024
	*ARMC8*	−0.5759	0.0196	0.025
	*PLEKHA8*	−0.5341	0.0331	0.039
	*BCL6B*	−0.5266	0.0361	0.039
	*API5*	−0.5109	0.0431	0.043
hsa-miR-1247-3p	*RHOBTB2*	−0.7882	0.0003	0.003
	*GRB7*	−0.7564	0.0007	0.004
	*KDSR*	−0.6962	0.0027	0.008
	*GLP1R*	−0.6809	0.0037	0.008
	*DVL1*	−0.677	0.004	0.008
	*AGFG2*	−0.6728	0.0043	0.008
	*EPN2*	−0.6434	0.0072	0.011
	*ACVRL1*	−0.5895	0.0162	0.022
	*WNT9B*	−0.5606	0.0239	0.028
	*NRP1*	−0.5567	0.0251	0.028
	*SEMA6D*	−0.5216	0.0383	0.038
hsa-miR-154-5p	*HECTD4*	−0.6384	0.0078	0.018
	*SALL1*	−0.6115	0.0118	0.018
	*C12orf29*	−0.5164	0.0406	0.041

The miRNA expression was evaluated by RT-qPCR (− ΔC_T_) and the respective mRNA expression was determined by RNA-sequencing (FPKM)

Since miRNAs down regulate their target mRNA/s expression, those target mRNAs in the RNA-seq dataset that exhibited the reverse pattern of expression compared to the miRNA expression pattern (negative correlation, *r* ≤ −0.5, *P* < 0.05) were selected. There was no significant correlation between the expression pattern of eca-miR-487b-3p and that of its predicted targets

**Fig. 4:**
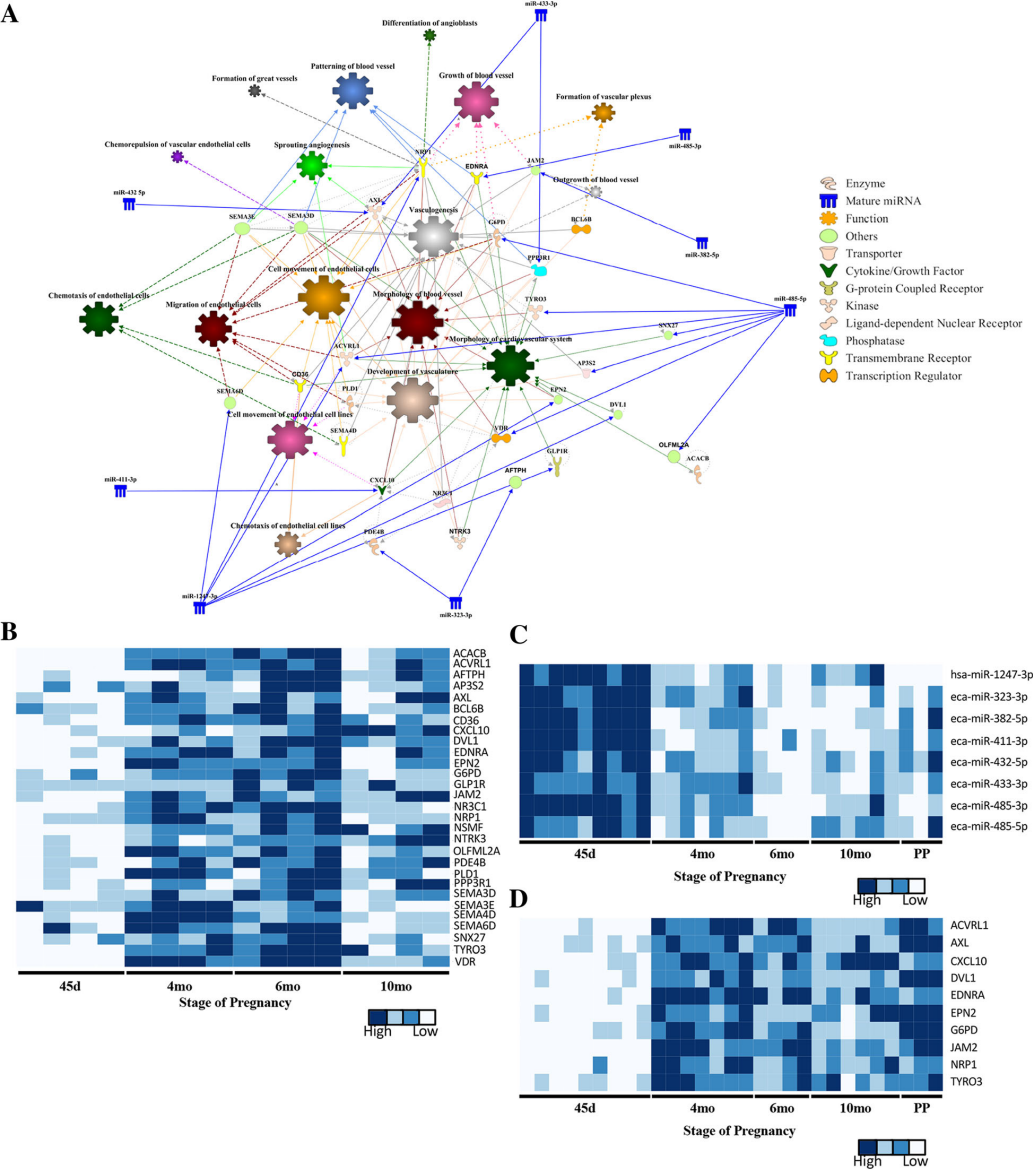
**a**. Pathway analysis of negatively correlated target mRNAs (*n* = 130) for differentially expressed C24MC-associated miRNAs presenting a decline in their expression during the course of gestation. Target mRNAs were mainly associated with cardiovascular development and function, and included endothelial cell movement/migration, angiogenesis, differentiation of angioblasts, formation of vascular plexus, and vascularization and morphology of blood vessels. Specific C24MC-associated miRNAs and direct relationships are shown in blue. Relevant physiological functions/pathways involved and their direct relationships are depicted with varying sizes according to the associated number of target mRNAs (ie. larger size indicative of higher number of mRNAs involved). The interactive figure can be accessed here. (http://rpubs.com/pouyadini/431871​) **b**. Expression patterns of mRNAs (*n* = 29 putative targets), which presented a significant negative correlation to their miRNA expression during gestation and are predicted to be involved in angiogenesis. The heatmap was constructed using FPKM values and scaled for each mRNA. The interactive heatmap can be accessed here. (http://rpubs.com/pouyadini/339221) **c**. Expression patterns of miRNAs (*n* = 8), which were predicted to be involved in angiogenesis in CAM during different stages of pregnancy (*n* = 29 samples). The heatmap was constructed using -ΔC_T_ values and scaled for each miRNA. The interactive heatmap can be accessed here. (http://rpubs.com/pouyadini/391422​) **d**. Expression patterns of putative mRNA targets involved in angiogenesis (*n* = 10) during the course of gestation in CAM (*n* = 29 samples). The heatmap was constructed using -ΔC_T_ values and scaled for each mRNA. The interactive heatmap can be accessed here. (http://rpubs.com/pouyadini/391679​)

**Fig. 5:**
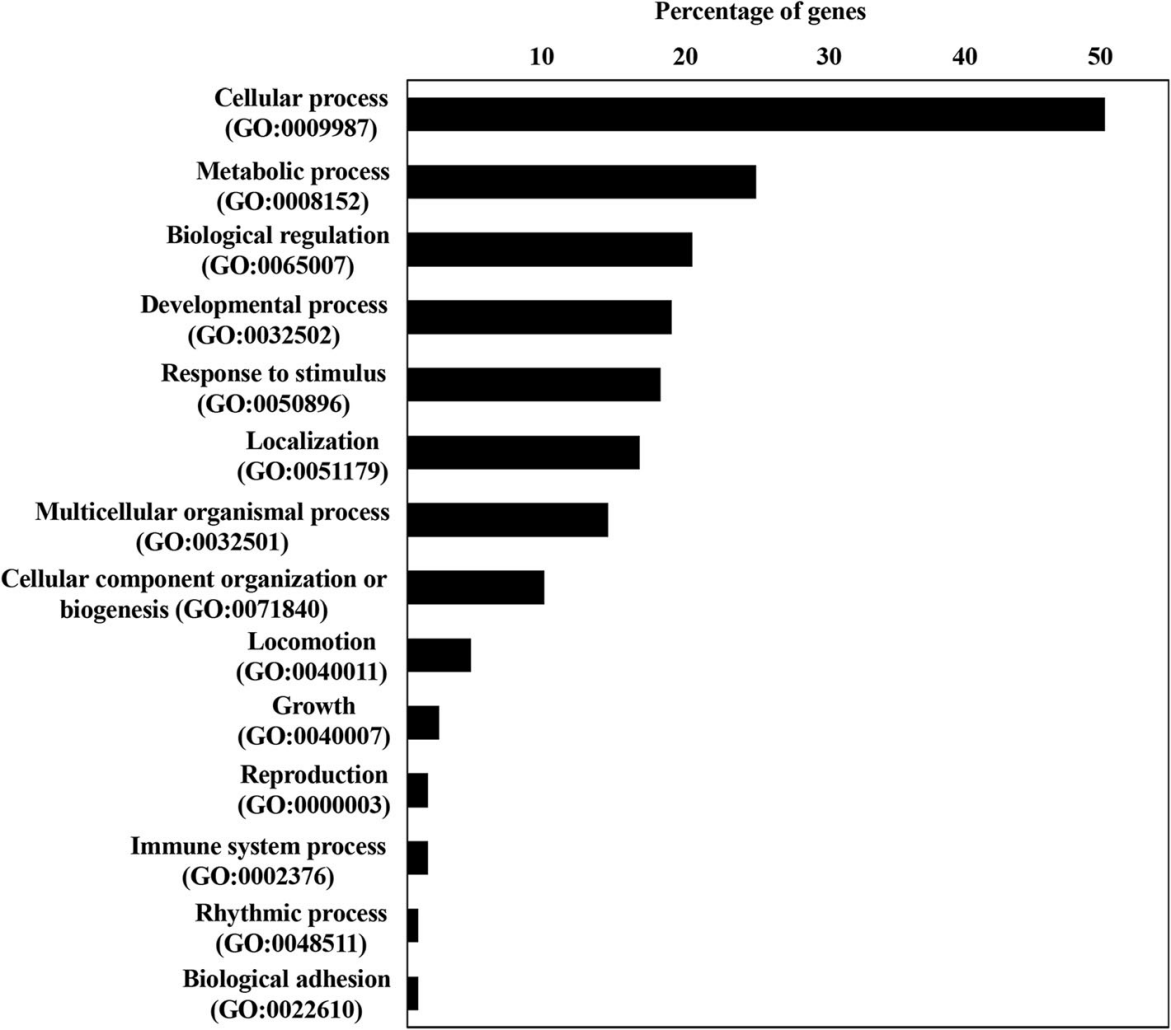
Functional annotation analysis of predicted mRNA targets using PANTHER

The physiological functions and canonical pathways for the target mRNAs were predicted using Ingenuity Pathway Analysis. The predicted physiological function analysis included involvement in cardiovascular development, embryonic development, tissue morphology and nervous system development and function (Table 5). Specific functions such as endothelial cell movement/migration, angiogenesis, differentiation of angioblasts, formation of vascular plexus, vascularization and morphology of blood vessels were among the annotated cardiovascular development processes (Fig. 4a). The expression pattern of the mRNAs and miRNAs involved in angiogenesis and vascular formation during gestation are presented in Fig. 4b and c, respectively. Interestingly, 31% (8/26) of the miRNAs evaluated were putatively associated with this specific biological function. In addition, immunosuppression and hypersensitivity/immediate hypersensitivity reactions were among the immunological functions assigned to the predicted targets. The complete list of functions and diseases associated with this subset of miRNAs can be found in Additional file 8: Table S8. The canonical pathways predicted for the target mRNAs primarily involved signaling pathways, including axonal guidance signaling, protein kinase A signaling, cAMP-mediated signaling, insulin-like growth factor 1 (IGF-1) and insulin receptor signaling, IL-4 signaling, and MAPK signaling, among others. The complete list of associated canonical pathways can be found in Additional file 9: Table S9. Gene ontology analysis of predicted mRNA targets was performed using PANTHER, and the biological processes mainly involved cellular (tyrosine kinase signaling pathway, MAPK cascade, cell proliferation, cycle and growth), metabolic (hydrolase activity and protein kinase activity), developmental (anatomical structure morphogenesis, cell differentiation, embryo development, ectoderm and mesoderm development) and immune system processes (receptor-mediated endocytosis and phagocytosis) (Fig. 5).

**Table 5:** The physiological function analysis prediction for the putative mRNA targets of C24MC-associated miRNAs with a significantly higher expression level during early stages of pregnancy (45d), followed by a decline toward the end of the gestation

Physiological Function		
	*P* value range	Number of genes involved
Cardiovascular System Development and Function	7.68E-03 - 2.63E-06	26
Tissue Morphology	8.79E-03 - 2.63E-06	49
Embryonic Development	8.99E-03 – 1.83E-05	36
Nervous System Development and Function	8.72E-03 – 2.18E-05	30
Behaviour	5.81E-03 – 4.50E-05	22

### Expression analysis of putative mRNA targets involved in angiogenesis

Since the top physiological function predicted for targeted mRNAs constituted cardiovascular system development (Table 5) and due to the importance of vessel formation in placenta development and function, we specifically selected a subset of genes (*n* = 10) participating from vascular morphology and cell movement (Fig. 4a and Additional file 8: Table S8) to confirm their expression by RT-qPCR during pregnancy in equine CAM (*n* = 29). These included *ACVRL1, AXL, CXCL10, DVL1, EDNRA, EPN2, G6PD, JAM2, NRP1* and *TYRO3*. There was a significant positive correlation between the expression pattern evaluated by RT-qPCR and RNA-seq data (i.e. -ΔC_T_ vs FPKM, 0.6 < *r* < 0.8 and *P* < 0.001). There was also a significant negative correlation between the expression pattern of miRNAs and their putative target mRNA (Table 6). The lowest expression level for these angiogenic-related genes was observed at 45d with a gradual increase in their expression level toward the end of gestation (Fig. 4d). Interestingly, their respective miRNAs presented the highest expression at 45d, decreasing thereafter (Fig. 4c). In addition, we evaluated the putative binding sites between the miRNAs and their putative mRNA targets (3’ UTR) and determined that these occur with a low free energy and, therefore, these miRNA:mRNA interactions are feasible (ΔG between − 12.6 and − 32.2; Table 7).

**Table 6:** Correlation analysis between selected target genes associated with angiogenesis and the respective members of C24MC in CAM during different stages of pregnancy (*n* = 29 samples)

Target mRNA	MIRNA ID	Correlation	*P* value
*ACVRL1*	hsa-miR-1247-3p	−0.72	< 0.0001
*ACVRL1*	eca-miR-485-5p	−0.54	0.0024
*AXL*	eca-miR-433-3p	−0.65	0.0001
*AXL*	eca-miR-432-5p	−0.55	0.0022
*CXCL10*	eca-miR-411-3p	−0.75	< 0.0001
*DVL1*	hsa-miR-1247-3p	−0.60	0.0006
*EDNRA*	eca-miR-485-3p	−0.60	0.0007
*EPN2*	hsa-miR-1247-3p	−0.53	0.0026
*G6PD*	eca-miR-485-5p	−0.58	0.0010
*JAM2*	eca-miR-382-5p	−0.59	0.0009
*NRP1*	hsa-miR-1247-3p	−0.58	0.0009
*TYRO3*	eca-miR-485-5p	−0.57	0.0012

**Table 7:** Putative mRNA:miRNA binding site and the free energy (ΔG)

Target mRNA	MIRNA ID	ΔG	3′ UTR binding site (5′ to 3′)	Position at 3′UTR	MIRNA seed sequence	Seed sequence position
*ACVRL1*	hsa-miR-1247-3p	−25.4	UCCUGGG	799-805	CCCGGGA	2-8
*ACVRL1*	hsa-miR-1247-3p	−20.6	CCUGGG	590-595	CCCGGG	2-7
*ACVRL1*	eca-miR-485-5p	−26.2	GCCAGCCUC	560-568	GAGGCUGGC	2-10
*ACVRL1*	eca-miR-485-5p	−23.1	AGCCUC	455-460	GAGGCU	2-7
*AXL*	eca-miR-432-5p	−25.3	UCUAAGA	878-884	UCUUCU	1-7
*AXL*	eca-miR-432-5p	−22.7	GCUCUAA	1073-1080	UUGGAGU	3-9
*AXL*	eca-miR-432-5p	−21.8	AUUCCAAGG	1245-1254	UCUUGGAGU	1-9
*AXL*	eca-miR-433-3p	−20.4	CAUGAU	495-502	AUCACG	1-6
*CXCL10* ^a^	eca-mR-411-3p	–	–	–	–	–
*DVL1*	hsa-miR-1247-3p	−32.2	CUCGGGG	441-447	CCCGGG	1-7
*EDNRA*	eca-miR-485-3p	−12.6	UGUGAC	100-105	GUCAUA	1-6
*EPN2*	hsa-miR-1247-3p	−27.4	CCGGGG	331-336	CCCGGGA	1-6
*EPN2*	hsa-miR-1247-3p	−26.9	CCUGGGG	2087-2093	CCCCGGGA	1-7
*EPN2*	hsa-miR-1247-3p	−25.4	UCUGGGG	366-372	CCCCGGGA	1-7
*G6PD*	eca-miR-485-5p	−27.9	CCAGCCUC	409-416	GAGGCUGG	2-9
*G6PD*	eca-miR-485-5p	−23.2	GGCCUC	99-105	GAGGCU	2-7
*G6PD*	eca-miR-485-5p	−20.9	GUCUGUCUCU	35-44	AGAGGCUGGC	1-10
*G6PD*	eca-miR-485-5p	−16.4	CCAGUUUC	514-521	GAGGCUGG	2-9
*JAM2*	eca-miR-382-5p	−20.4	GAGCAAUU	1131-1155	AGUUGUUC	3-10
*NRP1*	hsa-miR-1247-3p	−29.5	CCCGGG	873-879	CCCGGG	2-7
*NRP1*	hsa-miR-1247-3p	−21.5	CCCGGG	821-826	CCCGGG	2-7
*TYRO3*	eca-miR-485-5p	−18	AGUCUCU	899-905	AGAGGCU	1-7

^a^Binding site and free energy estimation not provided due to the lack of annotation of the 3′ UTR in the current equine genome assembly

### Localization of eca-miR-409 by in situ hybridization

**Fig. 6:**
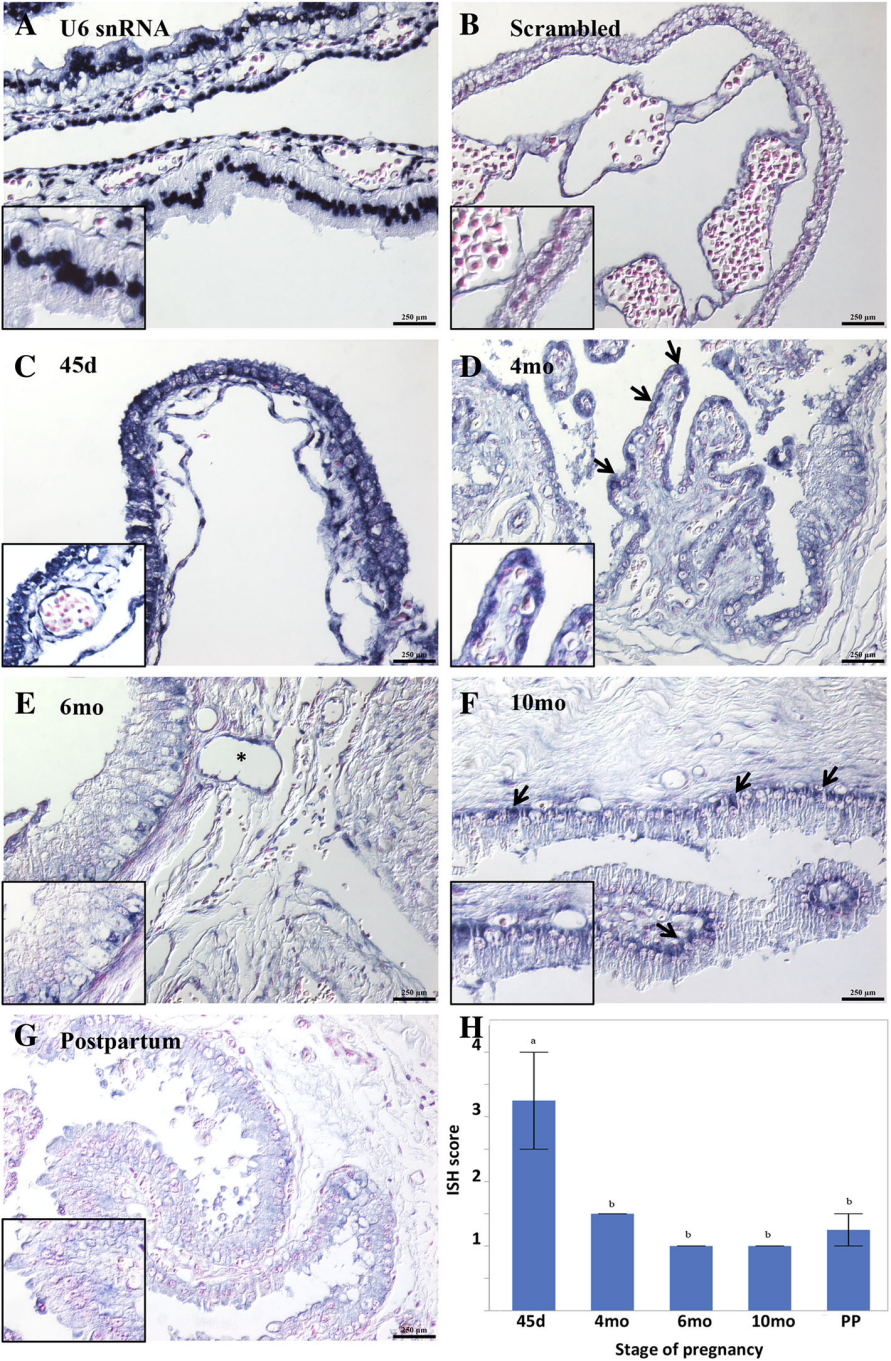
Localization of miR-409 in CAM samples during the course of gestation by in situ hybridization. NBT/BCIP (blue) was used as the substrate. Magnification: 200X. Bar = 250 μm. Insets represent a higher magnification (400X). **a** U6 snRNA control probe exhibits nuclear localization. **b** Scrambled miRNA probe (negative control). **c** CAM collected at 45d shows strong cytoplasmic signal for miR-409 specifically in chorioallantoic epithelium, vascular endothelium and mesenchymal cells. **d** and **e** CAM collected at 4mo and 6mo show a significant reduction in the expression of miR-409. MiR-409 expression was limited to scattered chorionic epithelial cells (arrows [**d**]), and rare mesenchymal and vascular endothelial cells (* [**e**]). **f** CAM collected at 10mo shows scattered, basolateral expression of miR-409 in chorionic epithelial cells (arrows) while no expression was observed in mesenchymal cells. **g** miR-409 expression was not detected in CAM samples collected postpartum. **h** The bar graph shows the mean value for each ISH score with standard error bars in CAM during the course of gestation. Mean miR-409 ISH scores between the two independent observers were determined, and mean scores were compared between the different stages of pregnancy

In order to study the cellular and subcellular localization of one member of C24MC (eca-miR-409-3p) in CAM, we performed miRNA in situ hybridization (ISH) on CAM sections during five different stages of pregnancy. Since eca-miR-409-3p had a differential expression pattern during different stages of pregnancy as determined by RT-qPCR analyses and presented a 100% homology to hsa-miR-409-3p, this miRNA was chosen as the best candidate for ISH analysis. MiR-409-3p signal was significantly higher at 45d (mean score of 3.25, *P* = 0.002), and its expression showed a decrease towards the end of pregnancy (Fig. 6c-h). The lowest miR-409-3p signal was observed at 6mo and 10mo (mean scores of 1, Fig. 6e-f and h). There was a significant correlation between -ΔC_T_ values and mean ISH scores (ρ = 0.9, *P* < 0.01). At 45d, miR-409-3p signal was predominantly observed in the cytoplasm of the chorionic and allantoic epithelium (Fig. 6c). MiR-409-3p was also abundantly expressed in mesenchymal cells and vascular endothelium (Fig. 6c). As indicated above, a significant reduction in miR-409-3p expression was observed at 4mo, 6mo and 10mo as well as in postpartum samples. At 4mo, positive signal was observed in scattered chorionic epithelial cells, which presented a mild intensity staining (Fig. 6d). Low signal was observed in the vascular endothelium, allantoic epithelium and scattered mesenchymal cells. At 6mo and 10mo, a low expression of miR-409-3p was observed in the chorionic epithelium, with basolateral expression in scattered epithelial cells specifically at 10mo (Fig. 6e, f). Finally, low miR-409-3p expression was observed in the vascular endothelium of scattered blood vessels, while no signal was detected in mesenchymal cells after 6mo.

## Discussion

In the present study, for the first time, we comprehensively and comparatively evaluated the kinetics of the equine C24MC (26 miRNAs), the ortholog of human C14MC, in the normal chorioallantoic membrane during the course of gestation. The chromosome 14 miRNA cluster, also referred to as the Mirg cluster [19], the miR-379/miR-410 cluster [51] or the miR-379/miR-656 cluster [13], is one of the largest identified miRNA clusters [14]. This cluster is located at the imprinted domain on the human 14q32 chromosomal interval (mouse chromosome 12 and equine chromosome 24) and abundantly expressed in the normal placenta [14]. By evaluating all sequenced genomes of mammals, it has been shown that this cluster is preserved without significant structural changes [13]. For example, all human C14MC miRNAs are conserved in the equids with the exception of five (hsa-miR-154, hsa-mir-300, mir-654, hsa-mir-665 and hsa-mir-668). Although human and murine C14MC and C12MC do not encode miR-3958 and miR-3959, these miRNAs have been predicted in equine C24MC and ovine C18MC orthologous clusters. This is the first report to unequivocally confirm the expression of the predicted eca-miR-3958-3p and eca-miR-3958-5p in equine CAM tissues. Here, we determined a significantly higher expression level of these miRNAs at 45d followed by a decline with increasing gestational age, demonstrating that these miRNAs have a similar expression pattern as other members of C24MC. Since current search databases for the identification of mRNA targets are based solely on human and murine miRNA annotation, prediction of eca-miR-3958-5p specific mRNA targets could not be performed. Currently, their predicted mRNA targets remain unknown and, therefore, identification of these targets is required to gain further insight into their functional role during gestation.

In previous studies, it has been shown that C14MC in humans is expressed predominantly during the first trimester of pregnancy and that its expression decreases as pregnancy advances [11, 23]. Morales-Prieto et al. analyzed the expression of C14MC in human cytotrophoblast cells collected from healthy first trimester placenta and term placenta (collected from postpartum samples) and showed that the expression of miRNAs within C14MC decreases significantly from first to third trimester [11]. This conclusion was based on a decline in expression levels of 16 miRNAs at only two time points. A recent study also observed the same expression pattern for six miRNAs within this cluster in villous tissue from human placenta obtained at the first trimester of pregnancy and postpartum placenta [23]. In the present study, we examined the expression kinetics of C24MC-associated miRNAs at five gestational time points in the entire CAM. Similar to human C14MC, we determined that the majority of the evaluated miRNAs (21/26) were highly expressed at the beginning of pregnancy followed by a decline towards the end of pregnancy. Accordingly, we observed a similar pattern of expression for miR-409-3p, a C24MC member, by ISH and we determined the cellular localization of this miRNA within CAM during the course of gestation. It is important to notice that, although some miRNA sequences from this cluster overlap with specific genes in the opposite DNA strand, the high RIN values in the samples evaluated determine the reliability of the miRNA expression analysis performed herein. This is further supported by the fact that ongoing studies in our laboratory to elucidate the interplay between eca-miR-127 and RTL1, encoded in opposite DNA strands, demonstrated that the expression of these exhibit a negative correlation during pregnancy whereby expression of eca-miR-127 decreases throughout gestation while that of RTL1 increases (Dini and Ball, 2018, unpublished).

Several studies have been published on the importance and functions of miRNAs on placental performance [52, 53]. For example, by restricting genes encoding critical miRNA biosynthetic enzymes such as Dicer [54] or Ago2 [55, 56], normal placental function was impaired, leading to early post-implantation embryonic lethality in mice. Others have used more targeted murine gene knockout experiments to elucidate miRNA functions in the placenta [57]. Even though these experimental functional analysis are available, they are limited to small number of miRNAs and target genes [35]. The findings presented herein suggest that the differential expression of several miRNAs within C24MC may be associated with placental development and performance during the course of gestation. In that regard, experimentally evaluating their functional role in vivo or in vitro constitutes a challenging task, particularly for a cluster of miRNAs. Thus, computational prediction of miRNA targets is the main tool for identifying possible miRNA:mRNA interactions and miRNA functions when a group of miRNAs are studied simultaneously [35, 58]. Computational algorithms can generate a list of putative genes targeted for the miRNA of interest; however, the presence of these genes in the specific cell or tissue of interest is not guaranteed. Another drawback of computational target predictions involves the occurrence of false putative targets (false positives). It has been shown that most of the target prediction algorithms have false positive rates of several tens of percent and, thus, may compromise functional inferences [59–61]. In the current study, to assess the predicted targets and have an insight into the functional role of C24MC-associated miRNAs in CAM, we generated an RNA-seq dataset from the same sample set to characterize the specific transcriptome profile of equine CAM throughout pregnancy as an alternative approach to specifically identify our predicted miRNA-targeted genes in the tissue of interest (CAM) with a higher confidence. Initially, we confirmed the presence of predicted target mRNAs in CAM tissue, followed by extensive correlation analysis between the expression profile of each miRNA with that of its predicted target mRNAs throughout pregnancy. It is well-known that miRNAs downregulate their target mRNA expression and, therefore, the expression pattern of specific miRNAs and that of their targets tend to be negatively correlated [36, 37]. We analyzed the negative correlation for the predicted targets and were able to narrow the list of putative targets to 130 genes (out of 1975 initially predicted). We believe this approach provided us with a more representative putative target list for C24MC in equine CAM.

Several functions have been proposed for this cluster including immune suppressive functions, participation in the anti-inflammatory response, protection from the ischemic/hypoxia injury, cell motility and participation in angiogenesis [10, 11, 51, 62]. It has been shown that C14MC has a pivotal effect on the feto-maternal interface of the placenta specifically on development of the endothelial and trophoblast cell interaction [19, 63, 64]. Mice with knockout C14MC had severe abnormalities associated with fetal capillaries in the placenta [63]. This capillary abnormality leads to overgrowth of the placenta, due to the large number of vacuoles in endothelial cells and the surrounding trophoblast layer of the fetal capillaries in the labyrinth zone causing neonatal lethality [63]. Interestingly, it has been shown that disruptions in the expression of this cluster in tissues other than placenta are related to vascular invasion [65]. In line with this, our computational target prediction analysis suggested that miRNAs from the C24MC target specific mRNAs involved in angiogenesis, angiogenesis of tissue of epithelial origin, vascularization, and development and migration of endothelial cells. Also, in another knockout model in mice, it has been shown that murine C12MC plays a number of roles required for neonatal survival [19]. Additionally, in vitro studies have shown that several miRNAs regulate trophoblast cell proliferation, invasion, and angiogenesis [66]. Consequently, the changes in the expression of C24MC-associated miRNAs during the course of pregnancy suggest that this may lead to modifications in angiogenesis within the placenta as pregnancy advances. For instance, it has been shown that *CXCL10*, which is a target gene for miR-411, can modify the development of newly formed vasculature [67]. We hypothesize that the reduction in the expression of some of the miRNAs in this cluster triggers the upregulation of angiogenesis-related genes that allow the process of angiogenesis to occur as the placenta develops. Here, we investigated the expression pattern of 10 selected putative mRNAs including *CXCL10* during the course of gestation and demonstrated a negative correlation with the respective miRNAs targeting this set of genes. For instance, the low expression of these mRNAs at 45d was accompanied by a high expression of their respective miRNAs. Furthermore, estimation of the miRNA binding sites at the 3’ UTR of their respective (predicted) mRNA targets demonstrated that all of the putative miRNA:mRNA interactions occur with a low free energy, which is indicative that these RNA:RNA interactions are feasible. Even though these results suggest the potential influence of these miRNAs in the regulation of this set of angiogenic genes, further in vivo or in vitro studies to elucidate the effect of these miRNAs on angiogenesis during placental development are warranted.

Finally, we localized eca-miR-409-3p in CAM and compared its expression during different stages of pregnancy. Our data suggest that the expression level and localization of this C24MC-associated miRNA is dependent upon the developmental stage of the CAM. While eca-miR-409 expression was high in the chorion and allantoic epithelia, mesenchymal cells and vascular endothelium in early pregnancy (45d), its expression was significantly reduced after 4mo with gradual loss of expression in these cell types. The downregulation of this miRNA in vascular endothelial cells along with an increase in the expression of angiogenic genes further suggests that this miRNA cluster is likely involved in the regulation of angiogenesis during placental development. Moreover, there was a significant correlation between expression levels of this specific miRNA determined by RT-qPCR and ISH. This is the first report on the expression of one of the C24MC members at the cellular and subcellular level in CAM throughout the pregnancy. The differential expression pattern of this miRNA suggests its role during pregnancy is dynamic and dependent on the stage of gestation.

## Conclusion

In conclusion, for the first time, we have comprehensively evaluated the kinetics of the C24MC in CAM, orthologous to the human C14MC, during equine pregnancy. We demonstrated that the expression of C24MC-asssociated miRNAs decreased during the course of gestation. We generated an RNA-seq dataset from equine CAM during pregnancy and predicted the functions of C24MC-associated miRNAs in this tissue. We have correlated the expression profile of C24MC-associated miRNAs and targeted mRNAs to evaluate the role of this cluster in the modulation of gene expression during placental development in vivo. We observed an inverse expression pattern for the putative target mRNAs when compared to the respective miRNA profile. Predictive pathway analysis indicated that members of C24MC targeted genes relevant to angiogenesis/vascularization, a process that has an active role during placental development and function. Finally, we successfully localized and evaluated the expression kinetics of a member of this cluster in specific CAM cells during equine pregnancy. Thus, this study provides fundamental information regarding the C24MC kinetics during pregnancy that will aid to further understand its role during equine gestation. Future studies to functionally understand the importance of this miRNA cluster in modulating gene expression during placental development are warranted.

## Additional files

Additional file 1: **Table S1.** Comparative list of C14MC associated-miRNAs among different species. The comparative list of C14MC in domestic animals. (XLSX 13 kb)

Additional File 2: Brief methodology for miRNA-sequencing, including mapping, normalization and statistical analysis. (PDF 64 kb)

Additional file 3: **Table S3.** Normalized read count for C24MC-associated miRNAs generated with Illumina miRNA-sequencing. (XLSX 24 kb)

Additional file 4: **Table S4.** Differential expression analysis results for C24MC-associated miRNAs during gestation, generated with Illumina miRNA-sequencing. (XLSX 21 kb)

Additional file 5: **Table S5.** Primer sequences for genes which were indicated in our dataset to be involved in angiogenesis. (XLSX 10 kb)

Additional file 6: **Table S6.** Read counts and mapping quality of Illumina RNA-sequencing dataset from CAM at different stages of pregnancy. Information about RNA-sequencing dataset. (XLSX 12 kb)

Additional file 7: **Table S7.** List of putative target mRNAs for analyzed miRNAs and their expression values (FPKM) during different stages of pregnancy. (XLSX 325 kb)

Additional file 8: **Table S8.** List of disease and physiological functions predicted for C24MC-associated miRNAs with IPA. (XLS 127 kb)

Additional file 9: **Table S9.** List of canonical pathways predicted for C24MC-associated miRNAs with IPA. (XLS 41 kb)

## Abbreviations

10mo: 10 Months
45d: 45 Days
4mo: 4 Months
6mo: 6 Months
ACTB: Actin Beta
ACVLR1: A Receptor Like Type 1
Ago2: Argonaute 2
AXL: AXL Receptor Tyrosine Kinase
C12MC: Chromosome 12 microRNA Cluster
C14MC: Chromosome 14 microRNA Cluster
C19MC: Chromosome 19 microRNA Cluster
C24MC: Chromosome 24 microRNA Cluster
CAM: Chorioallantoic Membrane
CT: Cycle Threshold
CXCL10: C-X-C Motif Chemokine Ligand 10
DIG: Dual Digoxigenin
DVL1: Dishevelled Segment Polarity Protein 1
ENDRA: Endothelin Receptor Type A
EPN2: Epsin 2
FPKM: Fragments Per Kilobase of transcript per Million mapped reads
G6PD: Glucose-6-phosphate Dehydrogenase
GAPDH: Glyceraldehyde-3-Phosphate Dehydrogenase
GUSB: Glucuronidase Beta
IGF-1: Insulin-Like Growth Factor 1
IPA: Ingenuity Pathway Analysis
ISH: In Situ Hybridization
JAM2: Junction Adhesion Molecule 2
miRNA: MicroRNA
mRNA: Messenger RNA
ncRNAs: Non-Coding RNAs
NRP1: Neuropilin 1
RNA-seq: RNA Sequencing
TPS: Target Prediction Score
TYRO3: TYRO3 Protein Tyrosine Kinase 3
ΔC_T_: Delta C_T_
ΔG: Free Energy
