# Supplementary material for: Kinetics of the chromosome 14 microRNA cluster ortholog and its potential role during placental development in the pregnant mare
Source: BMC Genomics. 2018 Dec 20;19:954. doi: 10.1186/s12864-018-5341-2 (PMC6302407; doi:10.1186/s12864-018-5341-2)
Supplement: Supplementary file 2 — Brief methodology for miRNA-sequencing, including mapping, normalization and statistical analysis. (PDF 64 kb) [file 12864_2018_5341_MOESM2_ESM.pdf]

Additional File 2

A miRNA sequencing dataset was generated by Illumina miRNA-sequencing from chorioallantoic membrane (CAM) from pregnant (4 and 10 mo), and postpartum mares.

RNA Isolation and Library Preparation

Isolation of RNA from CAM was performed using RNeasy Mini Kit (Qiagen, Gaithersburg, MD, USA) per manufacturer's instructions. After extraction, RNA was analyzed by NanoDrop® (Thermo Fisher Scientific) and Bioanalyzer® (Agilent, Santa Clara, CA, USA) to evaluate concentration, purity and integrity. All samples had a 230/260 ratio > 1.8, a 260/280 ratio > 2.0 and an RNA integrity number > 8.0.

Library preparation was performed by the 3' and 5' adapter ligation. Complementary DNA (cDNA) was generated from the ligated product with SuperScriptIII reverse transcriptase (Thermo Fisher Scientific) using primer complimentary to the 3' adapter sequence. The resulting cDNA was amplified with Illumina universal primer and indexed-primer. Library size-selection was performed using a PippinHT instrument (Sage Science, Boston, MA, USA) with either a 3% agarose cassette or with 6% polyacrylamide gel. The library with proper insert size was sequenced with the NextSeq 500 as 80 bp single-end reads (Illumina, San Diego, CA).

Small RNASeq Data analysis

The raw FASTQ files for each sample were trimmed three times to remove the adaptor sequences and low-quality sequences with TrimGalore (Babraham Institute, Cambridge, UK). The data were mapped to the equine-specific miRNAs in miRBase (release 21) using sRNAanalyzer (http://srnanalyzer.systemsbiology.net)(Kozomara and Griffiths-Jones, 2011, 2014). All reads were normalized by log(((read count * 1,000,000)/total reads count per sample)+1). Differential expression was analyzed using one-way ANOVA, with significance set at $P < 0.01$.
